# Association of diabetes mellitus with decline in ankle-brachial index among patients on hemodialysis: A 6-year follow-up study

**DOI:** 10.1371/journal.pone.0175363

**Published:** 2017-04-13

**Authors:** Szu-Chia Chen, Mei-Yueh Lee, Jiun-Chi Huang, Hsiu-Chin Mai, Po-Lin Kuo, Jer-Ming Chang, Hung-Chun Chen, Yi-Hsin Yang

**Affiliations:** 1 Graduate Institute of Clinical Medicine, College of Medicine, Kaohsiung Medical University, Kaohsiung, Taiwan; 2 Division of Nephrology, Department of Internal Medicine, Kaohsiung Medical University Hospital, Kaohsiung Medical University, Kaohsiung, Taiwan; 3 Department of Internal Medicine, Kaohsiung Municipal Hsiao-Kang Hospital, Kaohsiung Medical University, Kaohsiung, Taiwan; 4 Faculty of Medicine, College of Medicine, Kaohsiung Medical University, Kaohsiung Medical University, Kaohsiung, Taiwan; 5 Division of Endocrinology and Metabolism, Department of Internal Medicine, Kaohsiung Medical University Hospital, Kaohsiung Medical University, Kaohsiung, Taiwan; 6 Department of Nursing, Kaohsiung Municipal Hsiao-Kang Hospital, Kaohsiung Medical University, Kaohsiung, Taiwan; 7 Institute of Medical Science and Technology, National Sun Yat-Sen University, Kaohsiung, Taiwan; 8 Department of Internal Medicine, Kaohsiung Municipal Cijin Hospital (Operated by Kaohsiung Medical University), Kaohsiung, Taiwan; 9 Division of Medical Statistics and Bioinformatics, Department of Medical Research, Kaohsiung Medical University Hospital, Kaohsiung Medical University, Kaohsiung, Taiwan; 10 School of Pharmacy, Kaohsiung Medical University, Kaohsiung Medical University, Kaohsiung, Taiwan; Nagoya University, JAPAN

## Abstract

Peripheral artery occlusive disease is common among diabetes mellitus (DM) and end-stage renal disease patients, and tends to progress faster and lead to worse outcomes. This study compared the association of DM with the decline in ankle-brachial index (ABI) among patients on hemodialysis (HD). This was a longitudinal analysis of ABI in HD patients from 2009 to 2015. Medical records and yearly ABI values were obtained. A longitudinal mixed-model analysis was used to evaluate ABI changing trends while accounting for within-patients correlation. There were 296 patients on HD in the period of 2009–2015. In a 6-year follow-up, those with DM had a more rapid ABI decline compared to non-DM patients (slopes: -0.014 vs. 0.010 per year, interaction *p* < 0.001). In DM patients, female sex, high pulse pressure, high triglyceride, low creatinine, and high uric acid were associated with a decrease in ABI. In non-DM patients, old age, high pulse pressure, high low-density lipoprotein cholesterol, and high uric acid were associated with a decreased in ABI. There were 49.6% of patients with a normal ABI experienced a decrease at least 0.1 of ABI from baseline, and 35.3% had a final ABI < 0.9 in patients with a baseline ABI ≥ 0.9 (n = 232). In this study, DM patients on HD tend to develop a more rapid decline in ABI than non-DM patients on HD. Age, sex, pulse pressure, lipid profile, creatinine, and uric acid are associated with a decreased in ABI.

## Introduction

The incidence of non-traumatic lower-extremity amputation among patients with end-stage renal disease (ESRD) is high and the most common indication is peripheral artery occlusive disease (PAOD) [1]. Moreover, among patients with ESRD, PAOD is associated with increased cardiovascular mortality, morbidity, and hospitalization [2]. The ankle-brachial index (ABI) is a reproducible, non-invasive index used to screen and detect PAOD, with 90% sensitivity and 95% specificity [3]. Ono et al.[4] used ABI to evaluate all-cause and cardiovascular mortality in patients on hemodialysis (HD) and found that a reduction in ABI predicted poor survival. The hazard ratios of ABI < 0.9, ≥ 0.9 to < 1.0, and ≥ 1.0 to < 1.1 for all-cause mortality were 4.04, 3.24, and 1.92, respectively. Even those with modest reductions in ABI (≥ 0.9 to < 1.1) had increased risk of all-cause and cardiovascular mortality^4^. Thus, identifying ESRD patients with decreasing ABI for aggressive treatment is important for attenuating the disease and improving survival.

Diabetes mellitus (DM) is the leading cause of chronic kidney disease worldwide, accounting for approximately 45% of ESRD cases in the Taiwan population on dialysis. Among patients with DM, PAOD is especially common, with a three-fold increased risk compared to the general population [5]. Furthermore, PAOD also tends to progress faster and lead to worse outcomes in DM patients [6]. The American Diabetes Association recommends measuring ABI for asymptomatic patients aged ≥ 50 years and for younger patients with DM or other vascular risk factors, and repeating the measurement every 5 years if the first result is normal [7]. Approximately 20% DM patients had a significant decrease in ABI > 0.1 [8], which was higher than that seen in the general population [9,10].

However, data on trends in ABI changes in HD patients with DM are limited. A decline in ABI may serve as an important marker for adverse PAOD outcomes. This study compared the association of DM with the decline in annual ABI over 6 years among patients on HD, and investigated the associated factors.

## Patients and methods

### Study design and patients

This study was conducted in a dialysis clinic in southern Taiwan. The inclusion criteria were: (1) maintenance HD therapy for at least 3 months; (2) age > 20 years; and (3) regular intake of anti-hypertensive or oral hypoglycemic agents for at least 1 month. Those with atrial fibrillation, bilateral below-knee amputation and hospitalization or antibiotic treatment in the last 4 weeks were excluded. Patients with only one ABI measurement during the follow-up period were also excluded. A total of 296 patients were enrolled from August 2009 to August 2015. For patients with first HD at or before 2009, their baseline measures were started at the year of 2009; for patients entering the study during 2010~2015, their baseline measures were taken at the entering year.

Each HD session was performed for 3.5–4.5 hours, with a blood flow rate of 250–300 mL/min and dialysate flow of 500 mL/min. Blood samples were taken before and after HD to calculate for the Kt/V [11].

### Ethics statement

The study protocol was approved by the Institutional Review Board of Kaohsiung Medical University Hospital (KMUHIRB-E(I)-20150256). Written informed consent was obtained from the patients, and all clinical investigations were conducted according to the principles expressed in the Declaration of Helsinki. The patients also consented to the publication of the clinical details.

Demographic and medical data, including age, sex, and co-morbidities were obtained from medical records and patient interviews. Laboratory data was measured from fasting blood samples using an AutoAnalyzer (Roche Diagnostics GmbH, D-68298 Mannheim COBAS Integra 400). Blood samples were taken within 1 month of ABI measurement. The Kt/V was evaluated as a dialysis marker and determined according to the Daugirdas procedure [11]. Information on patient medication, including intake of aspirin, angiotensin-converting enzyme inhibitors, angiotensin II receptor blockers, and HMG-CoA reductase inhibitors (statins) during the study period was obtained from medical records.

### Assessment of ABI

The ABI was measured 10–30 minutes before HD using an ABI-form device that automatically and simultaneously measured blood pressure in both arms and ankles using an oscillometric method [12]. Briefly, occlusion and monitoring cuffs were placed tightly around the upper arm without blood access and on both lower extremities with the patient in a supine position. The ABI was calculated by the ratio of the ankle systolic blood pressure divided by the arm systolic blood pressure. After obtaining bilateral ABI values, the lower one was used for analysis. The ABI measurements were done for each patient every August.

### Statistical analysis

Data were expressed as percentages, mean ± standard deviation, or median (25^th^-75^th^ percentile) for duration of dialysis and levels of triglyceride and parathyroid hormone. A mixed-effect model analysis was used to evaluate ABI yearly changes between DM groups. This approach treated each ABI measure from each participant as a separate observation and was adjusted for within participant correlations. Subjects were treated as random effects so the analysis was adjusted to each individual’s own ABI levels. A first order autoregressive error structure was accounted for within-patient correlation. This model also explored the significance of risk factors at individual yearly measures for the ABI decline in DM and non-DM groups separately as well as between DM and non-DM groups using an interaction term of DM groups with years. Potential confounding factors were also included in the analysis model as covariates, which included age, sex, duration of dialysis, smoking history, hypertension, coronary artery disease, cerebrovascular disease, pulse pressure, laboratory data including albumin, triglyceride, total cholesterol, low-density lipoprotein (LDL) cholesterol, high-density lipoprotein cholesterol, hemoglobin, creatinine, total calcium, phosphorous, calcium-phosphorous product, uric acid, parathyroid hormone, Kt/V, and medications use. Survival curves for ABI decrease > 0.1 and progression to ABI < 0.9, in patients with a baseline ABI ≥ 0.9, were obtained using Kaplan-Meier estimates. Statistical significance was set at *p* < 0.05. Statistical analysis was performed using the SAS statistical package version 9.4 (SAS Institutes, Cary, NC, USA).

## Results

In the study period of 2009–2015, 296 patients were included for analysis. A total of 1412 ABI measurements were provided over the 6-year period. The frequency of ABI measures for each participant was 2–3 times (33.9%), 4–5 times (23.1%) and 6–7 times (43.1%). The characteristics of the study patients were shown in Table 1. In the 6-year period, 56 died, including 20 with baseline ABI < 0.9 (31.7%).

**Table 1:** Comparison of baseline characteristics between patients with and without diabetes mellitus (DM).

Characteristics	All (n = 296)	DM (n = 140)	Non-DM (n = 156)	*p*
First ABI year				0.002
2009 or before	177	69	108	
2010–2015	119	71	48	
Age at first ABI (year)	58.0 ± 11.8	60.8 ± 9.1	55.6 ± 13.4	< 0.001
Male gender (%)	51.0	54.3	48.1	0.286
Duration of dialysis (years)	2.5 (0.8–7.0)	1.3 (0.6–4.4)	5.1 (1.3–9.2)	< 0.001
Smoking history (%)	33.4	41.7	26.3	0.005
Hypertension (%)	71.2	80.0	63.5	0.002
Coronary artery disease (%)	18.9	22.9	15.4	0.101
Cerebrovascular disease (%)	7.8	13.6	2.6	< 0.001
Systolic blood pressure (mmHg)	151.6 ± 25.9	160.2 ± 25.4	143.9 ± 23.9	< 0.001
Diastolic blood pressure (mmHg)	80.7 ± 14.5	82.0 ± 14.0	79.6 ± 14.9	0.154
Pulse pressure (mmHg)	71.0 ± 17.6	78.5 ± 17.8	64.3 ± 14.5	< 0.001
Laboratory parameters				
Albumin (g/dL)	3.9 ± 0.3	3.9 ± 0.3	4.0 ± 0.3	0.001
Triglyceride (mg/dL)	125.5 (89.3–192)	140 (104–208)	119 (83–181)	0.006
Total cholesterol (mg/dL)	178.4 ± 42.2	182.5 ± 49.2	174.8 ± 34.5	0.135
HDL-cholesterol (mg/dL)	40.3 ± 11.3	38.0 ± 10.0	42.5 ± 12.0	< 0.001
LDL-cholesterol (mg/dL)	89.2 ± 31.3	91.8 ± 36.2	86.8 ± 26.0	0.182
Hemoglobin (g/dL)	10.1 ± 1.3	10.2 ± 1.2	10.0 ± 1.3	0.426
Creatinine (mg/dL)	10.2 ± 2.4	9.5 ± 2.7	10.7 ± 2.5	< 0.001
Total calcium (mg/dL)	9.3 ± 0.9	9.2 ± 0.8	9.4 ± 0.9	0.054
Phosphorous (mg/dL)	4.9 ± 1.3	4.9 ± 1.3	4.8 ± 1.4	0.620
Calcium-phosphorous product (mg^2^/dL^2^)	45.2 ± 12.7	45.1 ± 12.1	45.4 ± 13.2	0.840
Uric acid (mg/dL)	7.8 ± 1.6	7.7 ± 1.6	7.8 ± 1.6	0.572
PTH (pg/mL)	312.6 (160.7–532.7)	260.8 (150.5–421.6)	351.2 (173.6–633.2)	0.011
Kt/V (Daugirdas)	1.5 ± 0.3	1.5 ± 0.3	1.6 ± 0.3	0.035
Medications				
Aspirin use (%)	8.1	10.0	6.4	0.259
ACEI and/or ARB use (%)	16.2	21.4	11.5	0.021
Statin use (%)	16.6	21.4	12.2	0.033
ABI < 0.9 (%)	21.0	30.9	12.2	< 0.001
Death (%)	19.0	22.5	15.9	0.153

Abbreviations. ABI, ankle-brachial index; HDL, high-density lipoprotein; LDL, low-density lipoprotein; PTH, parathyroid hormone; ACEI, angiotensin converting enzyme inhibitor; ARB, angiotensin II receptor blocker.

### Association of baseline and change in ABI

**Fig 1:**
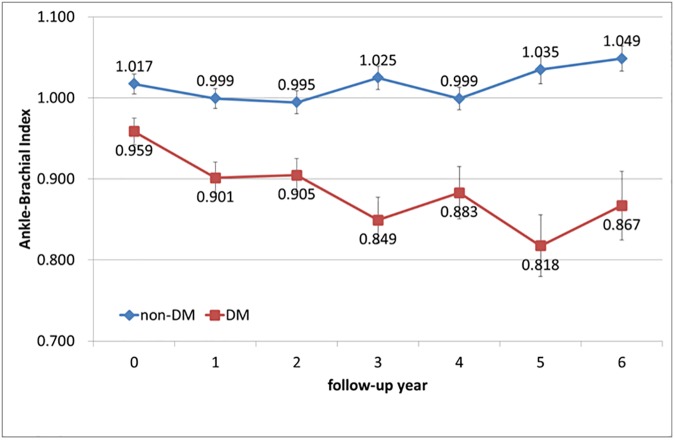
Mean Ankle-Brachial Index at each follow-up year for DM and non-DM patients (error bars indicate standard errors).

In terms of ABI changes in HD patients with or without DM, the ABI declined from 0.959 to 0.867 over 6 years in patients with DM but only declined from 1.017 to 1.049 in non-DM patients. The ABI decline curve by follow-up years among DM and non-DM patients (Fig 1) revealed that HD patients with DM had a rapid decline in ABI, and no decline in ABI in non-DM patients. Using the estimated slopes by a mixed-effect model showed -0.023 (95% confidence interval [CI], -0.032 to -0.014; *p* < 0.001) for DM patients and 0.002 (95% CI, -0.005 to 0.010; *p* = 0.535) for non-DM patients. A comparison of the two slopes by an interaction term indicated significant difference (-0.025, 95% CI, -0.038 to -0.013; *p* < 0.001) over the 6-year follow-up. When adjusting by all potential confounders in the mixed-effect model, the slopes of DM and non-DM as well as the coefficient of interaction were -0.014 (95% CI, -0.024 to -0.003; *p* = 0.010), 0.010 (95% CI, 0.001 to 0.019; *p* = 0.027) and -0.024 (95% CI, -0.036 to -0.012; *p* < 0.001), respectively.

We have further performed sensitivity analysis using ABI change from either right or left sides. Using the estimated slopes from using right side ABI measures showed -0.015 (95% CI, -0.025 to -0.005; *p* = 0.002) for DM patients and 0.002 (95% CI, -0.006 to 0.010; *p* = 0.681) for non-DM patients. A comparison of the two slopes by an interaction term indicated significant difference -0.017 (95% CI, -0.030 to -0.004; *p* = 0.009) over the 6-year follow-up. Besides, using the estimated slopes showed -0.024 (95% CI, -0.037 to -0.011; *p* < 0.001) for DM patients and 0.000 (95% CI, -0.008 to 0.008; *p* = 0.991) for non-DM patients from using left side. A comparison of the two slopes by an interaction term indicated significant difference -0.024 (95% CI, -0.037 to -0.011; *p* < 0.001) over the 6-year follow-up. These estimates were similar to the estimate from using the minimum of ABI.

### Determinants of ABI decline in HD patients with DM

Using the mixed-effect model, the main effects of the variables on ABI change in HD patients with DM are female sex (coefficient: 0.1099; 95% CI, 0.0363 to 0.1835; *p* = 0.004), high pulse pressure (coefficient: -0.0028; 95% CI, -0.0038 to -0.0019; *p* < 0.001), high triglyceride (coefficient: -0.1116; 95% CI, -0.2189 to -0.0043; *p* = 0.042), low creatinine (coefficient:0.0130; 95% CI, 0.0015 to 0.0245; *p* = 0.027), and high uric acid (coefficient: -0.0110; 95% CI, -0.0219 to 0; *p* = 0.049) were significantly associated with a decrease in ABI (Table 2).

**Table 2:** Main effects of the variables on ABI change over year in hemodialysis patients with DM.

Variables	ABI change	95% confidence intervals	*p*
Age (per 1 year)	-0.0024	(-0.0050, 0.0002)	0.072
Male *vs*. Female	0.1099	(0.0363, 0.1835)	0.004
Duration of dialysis (per 1 year)	-0.0058	(-0.0143, 0.0028)	0.185
Smoking history	-0.0645	(-0.1332, 0.0043)	0.066
Hypertension	0.0002	(-0.0515, 0.0519)	0.994
Coronary artery disease	-0.0186	(-0.0705, 0.0334)	0.483
Cerebrovascular disease	-0.0083	(-0.0716, 0.0549)	0.796
Pulse pressure (per 1 mmHg)	-0.0028	(-0.0038, -0.0019)	< 0.001
Laboratory parameters			
Albumin (per 1 g/dL)	-0.0316	(-0.1041, 0.0409)	0.392
Triglyceride (per log 1 mg/dL)	-0.1116	(-0.2189, -0.0043)	0.042
Total cholesterol (per 1 mg/dL)	0.0005	(-0.0002, 0.0012)	0.132
HDL-cholesterol (per 1 mg/dL)	0.0019	(-0.0006, 0.0043)	0.128
LDL-cholesterol (per 1 mg/dL)	-0.0005	(-0.0013, 0.0003)	0.183
Hemoglobin (per 1 g/dL)	-0.0084	(-0.0212, 0.0043)	0.194
Creatinine (per 1 mg/dL)	0.0130	(0.0015, 0.0245)	0.027
Total calcium (per 1 mg/dL)	0.0250	(-0.0359, 0.0858)	0.420
Phosphorous (per 1 mg/dL)	0.0430	(-0.0628, 0.1488)	0.425
Calcium-phosphorous product (per 1 mg^2^/dL^2^)	-0.0051	(-0.0164, 0.0061)	0.372
Uric acid (per 1 mg/dL)	-0.0110	(-0.0219, 0.0000)	0.049
PTH (per log 1 pg/mL)	-0.0026	(-0.0369, 0.0317)	0.880
Kt/V (per 1)	0.0353	(-0.0459, 0.1165)	0.393
Medications			
Aspirin use	-0.0356	(-0.0958, 0.0246)	0.245
ACEI and/or ARB use	-0.0323	(-0.0772, 0.0126)	0.158
Statin use	0.0174	(-0.0268, 0.0615)	0.440

Abbreviations are same as Table 1. Values expressed as ABI change and 95% confidence interval (CI).

### Determinants of ABI decline in HD patients without DM

The main effects of the variables on ABI change in HD patients without DM demonstrated that old age (coefficient: -0.0026; 95% CI, -0.0040 to -0.0012; *p* < 0.001), high pulse pressure (coefficient: -0.0009; 95% CI, -0.0017 to -0.0002; *p* = 0.019), high LDL-cholesterol (coefficient: -0.0011; 95% CI, -0.0021 to -0.0002; *p* = 0.015), and high uric acid (coefficient: -0.0094; 95% CI, -0.0170 to -0.0018; *p* = 0.015) were significantly associated with a decrease in ABI (Table 3).

**Table 3:** Main effects of the variables on ABI change over year in hemodialysis patients without DM.

Variables	ABI change	95% confidence intervals	*p*
Age (per 1 year)	-0.0026	(-0.0040, -0.0012)	< 0.001
Male *vs*. Female	0.0283	(-0.0115, 0.0681)	0.163
Duration of dialysis (per 1 year)	-0.0027	(-0.0057, 0.0003)	0.081
Smoking history	0.0253	(-0.0110, 0.0615)	0.171
Hypertension	-0.0138	(-0.0426, 0.0149)	0.345
Coronary artery disease	-0.0125	(-0.0599, 0.0350)	0.606
Cerebrovascular disease	-0.0051	(-0.0843, 0.0741)	0.900
Pulse pressure (per 1 mmHg)	-0.0009	(-0.0017, -0.0002)	0.018
Laboratory parameters			
Albumin (per 1 g/dL)	0.0252	(-0.0240, 0.0744)	0.315
Triglyceride (per log 1 mg/dL)	-0.0585	(-0.1383, 0.0212)	0.150
Total cholesterol (per 1 mg/dL)	0.0006	(-0.0002, 0.0014)	0.137
HDL-cholesterol (per 1 mg/dL)	-0.0001	(-0.0017, 0.0015)	0.885
LDL-cholesterol (per 1 mg/dL)	-0.0011	(-0.0021, -0.0002)	0.015
Hemoglobin (per 1 g/dL)	-0.0019	(-0.0109, 0.0070)	0.673
Creatinine (per 1 mg/dL)	-0.0030	(-0.0115, 0.0054)	0.482
Total calcium (per 1 mg/dL)	-0.0046	(-0.0446, 0.0354)	0.823
Phosphorous (per 1 mg/dL)	0.0004	(-0.0752, 0.0759)	0.993
Calcium-phosphorous product (per 1 mg^2^/dL^2^)	-0.0005	(-0.0085, 0.0075)	0.900
Uric acid (per 1 mg/dL)	-0.0094	(-0.0170, -0.0018)	0.015
PTH (per log 1 pg/mL)	0.0016	(-0.0217, 0.0250)	0.892
Kt/V (per 1)	0.0169	(-0.0384, 0.0721)	0.549
Medications			
Aspirin use	-0.0445	(-0.1011, 0.0121)	0.123
ACEI and/or ARB use	-0.0076	(-0.0436, 0.0284)	0.679
Statin use	0.0240	(-0.0093, 0.0574)	0.157

Abbreviations are same as Table 1. Values expressed as ABI change and 95% confidence interval (CI).

### A decrease in ABI by > 0.1 or a final ABI < 0.9

**Fig 2:**
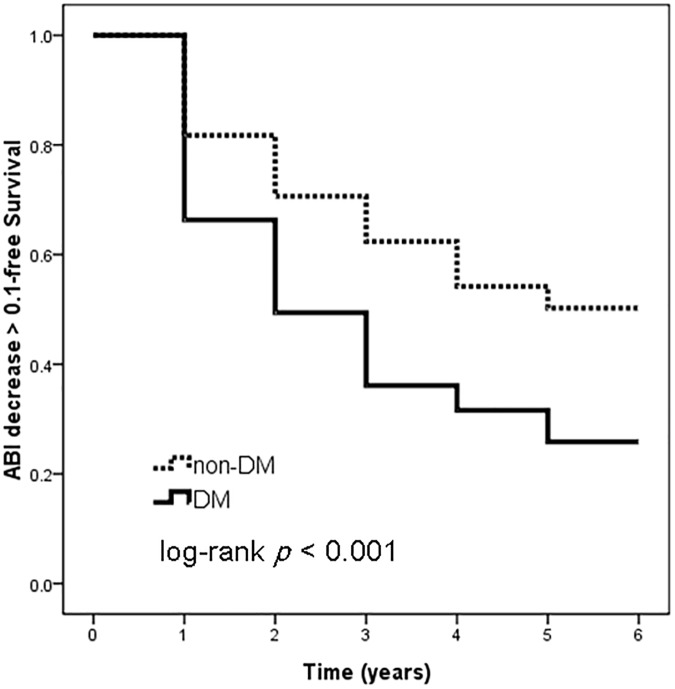
Kaplan-Meier analyses of ABI decrease > 0.1-free survival in patients with a baseline ABI ≥ 0.9. DM patients had worse ABI decrease > 0.1-free survival (log-rank test, *p* < 0.001) than non-DM patients.

**Fig 3:**
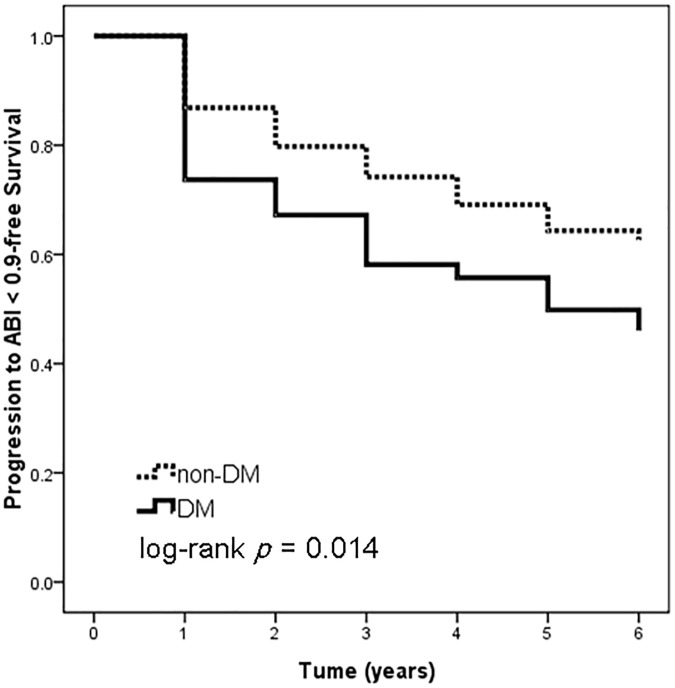
Kaplan-Meier analyses of progression to ABI < 0.9-free survival in patients with a baseline ABI ≥ 0.9. DM patients had worse progression to ABI < 0.9-free survival (log-rank test, *p* = 0.014) than non-DM patients.

We further perform subgroup analysis in patients with baseline ABI ≥ 0.9 (n = 232). There are 49.6% of patients with a normal ABI experienced a decrease at least 0.1 of ABI from baseline, and 35.3% have a final ABI < 0.9. Figs 2 and 3 illustrates the Kaplan-Meier survival curves for ABI decrease > 0.1 and progression to ABI < 0.9, respectively, in patients with baseline ABI ≥ 0.9. DM patients had worse ABI decrease > 0.1-free survival (log-rank test, *p* < 0.001) and progression to ABI < 0.9-free survival (log-rank test, *p* = 0.014) than non-DM patients.

## Discussion

There have been no published studies on serial measurements of ABI in HD patients. The present study is the first to evaluate changes in ABI yearly in HD patients over 6 years. We evaluated the associations of DM with decline in ABI yearly over 6 years in HD patients. The study was allowing us to describe the natural history of ABI in HD patients, and found the association of DM on decline in ABI. Age, sex, pulse pressure, lipid profile, creatinine, and uric acid were associated with decline in ABI in HD patients.

The most significant finding here is that HD patients with DM tend to portend a more aggressive course, with a rapid ABI decline, compared to non-DM patients (slopes: -0.014 vs. 0.010 per year, interaction *p* < 0.001) over a 6-year follow-up. Some studies have looked at the progression of PAOD in selected patient populations via serial measurements of ABI in two readings [8,13–16]. The study by Bird [14] surveyed ABI changes in 508 PAOD patients and found a mean ABI change of -0.019 over a 4.6-year average follow-up (about -0.004 per year). Similarly, Hoe et al.[8] assessed ABI change in 82 DM patients over a mean follow-up of 27.6 months and demonstrated that one in five DM patients had a significant decrease in ABI > 0.1 [8]. In addition, Jiwakanon et al.[16] evaluated ABI changes in 167 proteinuric DM patients after a mean interval of 23 ± 6 months and found that 17% of patients had either an ABI decrease of ≥ 0.1 or a final ABI < 0.9 [16]. Althouse et al.[13] likewise investigated ABI change for an average 4.6 years of follow-up in 1479 DM patients, and found that approximately 20% of participants with normal ABI had at least one PAOD-related incident. The PAOD-related incident included new ABI ≤ 0.9 and a decrease of at least 0.1 from baseline, lower extremity revascularization, or lower extremity amputation [13]. In HD patients, Chen et al. assessed the progression of PAOD and revealed a mean ABI change of -0.04 in 3 years (about -0.013 per year) [15]. In the present study, the mean ABI decrease in HD patients with DM is about -0.017 per year, significantly higher compared to the findings of previous studies (ABI decline rates of -0.004 to -0.013 per year) [14,15]. Besides, our study showed there were 49.6% of patients with a normal ABI experienced a decrease at least 0.1 of ABI from baseline, and 35.3% had a final ABI < 0.9, which frequency were also higher than previous studies [8,13,16]. There is growing evidence that uremia itself promotes PAOD progression [17,18]. Such processes may include vascular calcification, inflammatory and coagulation pathways alterations, oxidative stress, malnutrition, or infection [19,20].

Another important finding of the present study is that high triglyceride, and high LDL-cholesterol are associated with a rapid ABI decline among HD patients. In previous cross-sectional studies, the identified risk factors of PAOD in HD patients are old age, hypertension, DM, previous coronary artery disease or cerebrovascular disease, wider pulse pressure, hyperlipidemia, malnutrition, and smoking [18]. Some longitudinal studies also support the role of lipid profile in PAOD progression [8,9,13]. High triglyceride and LDL-cholesterol are major risk factors of atherosclerosis [21,22], which was consistent with our results. Lipid-lowering agents may be effective in the primary prevention of coronary artery disease [23], but there is no evidence that these drugs are effective in preventing or treating PAOD. In a meta-analysis of seven prospective randomized trials of lipid-lowering agents of patients with existing PAOD, there is no significant improvement in pain, ABI, or skin necrosis [24]. Nonetheless, despite the paucity of effective PAOD treatment, most clinicians still prescribe lipid-lowering agents because of their proven benefits of reducing coronary artery and cerebrovascular disease in patients on HD. This warrants further investigation in order to develop interventions that can slow the rapid progression of PAOD in high-risk populations. However, treatment with statins could potentially influence lipid parameters, and the effects of the variables of cholesterols and triglycerides are affected by medications. Therefore, the results related with these variables should be carefully estimated.

Approximately 45% of all Taiwanese dialysis patients with ESRD have DM, and it is the major cause of chronic kidney disease worldwide. In addition, the rate of elderly and diabetic patients receiving renal replacement therapy has dramatically increased in recent years, and they now account for a significant proportion of all patients undergoing dialysis [25,26]. The clinical outcomes of elderly patients with ESRD and diabetes would be expected to be worse compared to a younger population without diabetes [27,28]. In addition, functional activity and rehabilitation have been shown to be more severely affected in older patients with diabetes [29]. In large part, the differences in survival between patients with and without diabetes are attributable to poorer nutritional status in those with diabetes. Furthermore, a lower body mass index and malnutrition (due to dialysis-related protein loss, increased inflammation causing increased protein catabolism and a loss of appetite due to changes in taste and dietary restrictions) is associated with worse outcomes in patients with ESRD. [30,31]. The present study shows that low creatinine is associated with PAOD progression in DM, not in non-DM patients. Compared to non-DM patients, DM patients had older age, lower albumin and creatinine, which may partly explain the role of creatinine in rapid PAOD progression in DM patients. Traditional risk factors like high blood pressure, obesity, and hypercholesterolemia also play important roles in cardiovascular mortality of the general population. The concept of reverse epidemiology has recently suggested that low body mass index, low blood pressure, hypocholesterolemia, low creatinine, and low homocysteine level are associated with high cardiovascular and total mortality in patients with ESRD [32,33]. Low creatinine level may be related to malnutrition and can cause poorer outcome by accelerating atherosclerosis and worsening inflammation [34]. In addition, creatinine level is not only a marker of nutrition but also of muscle mass, which is related to physical activity and performance. Skeletal muscular dysfunction severely affects mobility and physical performance, and increasing the risk of fractures, disability, hospitalization and mortality [35]. Impaired physical function is a major complication in patients with ESRD, and it is associated with both a low quality of life and an increased risk of all-cause mortality in dialysis patients [36,37].

The third important finding of this study is that in HD patients, regardless with or without DM, high uric acid level is associated with a rapid ABI decline. The mechanism by which uric acid is associated with atherosclerotic disease remains unclear. Uric acid may increase platelet adhesiveness [38,39], or urate crystals may be associated with increased platelet lysis [40]. Uric acid may also play a role in the formation of free radicals and in oxidative stress [41,42]. Moreover, hyperuricemia per se can induce endothelial dysfunction by inhibiting the synthesis and release of nitric oxide [43]. The renin-angiotensin system in vascular endothelial cells (a hormonal vasoconstriction system) is also activated by elevated uric acid [44]. A previous experiment has confirmed that the solubility of monosodium urate falls sharply with decreasing temperature [45]. Local temperature may partially explain the clinically observed distribution of gouty tophi and gouty arthritis. Presumably, urate deposition in the lower extremity of patients with PAOD during cold temperatures and promote urate-related atherosclerosis. We also observed that high uric aicd level was associated with a decrease in ABI in HD patients. This may play a role in the clinically observed progression of atherosclerosis in HD patients with hyperuricemia.

We also found that the progression of PAOD was more strongly associated with the female patients with DM than the male patients with DM. Male sex is a known risk factor for most heart valve and vascular diseases, whereas females are known to be at higher risk of other disorders [46]. Cardiovascular diseases are the leading cause of death and disability in both men and women worldwide, but affect more women than men. Cardiovascular diseases are mostly caused by traditional risk factors, and whereas the effects of high blood pressure, overweight and obesity, and high cholesterol levels on cardiovascular outcomes are generally similar between men and women, the risk incurred by diabetes is significantly higher in women than in men [46,47]. Menopause plays a vital role in this difference, as it leads to adverse changes in cardiovascular structure due to hormonal changes. Oestrogens are known to be important modulators of lipid metabolism, inflammation and vascular homeostasis. Endogenous oestrogen plays a role in reducing the incidence of atherosclerotic vascular disease in premenopausal women with intact ovarian function, therefore the decrease in oestrogen production after menopause leads to an increase in the risk of cardiovascular diseases [47]. Women with ESRD are often associated with menstrual and fertility disorders due to disturbances in the endocrine system mediated by the kidneys [48]. This situation is characterized by an early decrease in the reserve of ovarian follicles, resulting in amenorrhea, infertility and the long-term reduction in estrogen and androgen [48]. In the present study, all of the women undergoing HD except for seven without DM still had menopause. Therefore, factors specific to women undergoing HD may be risk factors for the progression of PAOD.

The strength of this study was its prospective serial follow-up of annual ABI measurements in 6 years in patients on HD, a high-risk group for PAOD. Moreover, a longitudinal mixed-model with risk factors of potential confounders at individual yearly measure was used to reflect the actual value change during the follow-up period. This tested for associations while accounting for within-patient correlation. Nonetheless, this study also has some limitations. First, the effect of medications on ABI change was not evaluated because this study was not a clinical trial aimed to investigate the effects of medication, which is lacking cumulative exposure duration and defined daily dose. Treatment with statins could potentially influence lipid parameters. However, because of ethical reason, we did not hold any drugs at the time of ABI evaluation. To decrease the influence of drugs, we had added statins in the multivariate analysis. Second, other important factors affecting PAOD progression like inflammatory markers were not evaluated. During follow-up, there were also a significant number of deaths that more likely had lower ABI. Overall, the effect of biased results might have caused an underestimation of the magnitude of PAOD progression in this study. Lastly, the sensitivity of ABI measurements for detection of PAOD among patients with ESRD has not been tested. This technique is probably less sensitive than in the general population, because of the high prevalence of arterial calcification in this population, especially with DM [4,49]. Thus, estimates of PAOD prevalence based on ABI testing might underestimate the true prevalence of PAOD in the ESRD population.

In conclusion, in this study, HD patients with DM tend to have a more rapid ABI decline compared with non-DM patients after 6 years of follow-up. Female sex, high pulse pressure, high triglyceride, low creatinine, and high uric acid were associated with a rapid ABI decrease in DM patients, and old age, high pulse pressure, high LDL-cholesterol, and high uric acid in non-DM patients.

## Supporting information

S1 File Relevant data including ABI change. (XLS)
